# Building health system resilience in Romania: a consensus on policy priorities

**DOI:** 10.25122/jml-2025-0123

**Published:** 2025-12

**Authors:** Teodor Cristian Blidaru, Alina Ioana Forray, Dragos Garofil, Larisa Mezinu-Bălan, Oana Lazăr, Iulia Stoea, Cristian Vlădescu, Laurențiu Dașcă, Gratiela Iordache, Radu Iliescu, Raluca Sîmbotin, Otilia Anghel, Adina-Maria Voda, Decebal Mohîrță, Ecaterina Pitel, Guenadiy Vatachki, Valentina Deleanu, Iuliu Cocuz

**Affiliations:** 1 Carol Davila University of Medicine and Pharmacy, Bucharest, Romania; 2 Faculty of Medicine, Iuliu Hațieganu University of Medicine and Pharmacy, Cluj-Napoca, Romania; 3 Romanian National Health Insurance House, Bucharest, Romania; 4 National Institute for Health Services Management, Bucharest, Romania; 5 Faculty of Medicine, Titu Maiorescu University of Bucharest, Bucharest, Romania; 6 Arensia Exploratory Medicine, Bucharest, Romania; 7 National Authority of Quality Management in Health, Bucharest, Romania; 8 Faculty of Medicine, Grigore T Popa University of Medicine and Pharmacy, Iasi, Romania; 9 Merck Sharp & Dohme Romania SRL, Bucharest, Romania; 10 World Bank Romania, Bucharest, Romania; 11 Public Affairs Solutions, Bucharest, Romania; 12 Faculty of Medicine, Transilvania University of Brasov, Brasov, Romania; 13 National Transplant Agency, Bucharest, Romania; 14 Roche Romania, Bucharest, Romania; 15 George Emil Palade University of Medicine, Pharmacy, Science, and Technology, Targu Mures, Romania

**Keywords:** Romania, healthcare reform, health financing, health workforce, hospital care, health policy, consensus development, AI, Artificial Intelligence, ANDIS, National Agency for Infrastructure Development in Health, CTIS, Clinical Trial Information System, DRG, Diagnosis-Related Group, eHDSI, eHealth Digital Service Infrastructure, EHDS, European Health Data Space, HER, Electronic Health Record, EU, European Union, FHIR, Fast Healthcare Interoperability Resources, GDPR, General Data Protection Regulation, HL7, Health Level Seven, IT, Information Technology, LOINC, Logical Observation Identifiers Names and Codes, NCPeH, National Contact Points for eHealth, NHIH, National Health Insurance House, NUSSHIF, National Unique Social Health Insurance Fund, OECD, Organisation for Economic Co-operation and Development, PaRIS, Patient-Reported Indicator Surveys, PIAS, Health Insurance IT Platform, PNRR, National Recovery and Resilience Plan, SMO, Site Management Organization, SNOMED CT, Systematized Nomenclature of Medicine-Clinical Terms

## Abstract

Romania’s healthcare system struggles with the EU’s highest rates of treatable and preventable mortality and the lowest per capita health expenditure. Critical issues include unsustainable financing, an entrenched physician-centered model lacking institutional accountability, delayed digitalization, and inadequate clinical research capacity, necessitating structural and paradigmatic shifts. The objective of this study was to synthesize multi-stakeholder consensus recommendations for comprehensive reform of the Romanian healthcare system, focusing on financing, service delivery, and human resources. This manuscript details recommendations from a multi-stakeholder consensus conference organized in April 2025 by the Aspen Institute Romania. Participants addressed topics including financing, patient-centered hospital models, clinical research, health innovation, and the European Health Data Space (EHDS). Consensus recommendations include: stabilizing national health insurance funds (broadened contributions, multi-year budgets); shifting hospitals to patient-centered, institutionally accountable models (transparent allocation, digital integration, eradicating informal payments); equitable hospital reimbursement (unified tariffs); accelerated digital transformation (EHDS alignment, national Electronic Health Record); enhanced clinical trial capacity (personnel, infrastructure, regulatory efficiency); and exploring regulated dual practice, contingent on successful prior reforms. Key challenges include transforming hospital culture, promoting digital adoption, and navigating the complexities of politics and finance. These interconnected recommendations form a roadmap for transformative reform, crucial given the untenable status quo. Success requires sustained political will, stakeholder collaboration, investment, and robust governance to create a financially stable, patient-centered, equitable, and innovative system.

## INTRODUCTION

Following the collapse of communism in 1989, Romania transitioned from the centralized Semashko model to a Bismarck-style social health insurance system, which included the introduction of family medicine and mandatory health insurance in the 1990s [1-4]. Since becoming a member of the European Union (EU) in 2007, various national health strategies, such as the 2014–2020 plan and the ongoing 2023–2030 plan, have focused on upgrading the system by investing in health research, e-health technologies, and infrastructure [1, 5-8]. However, persistent challenges such as underfunding, workforce shortages, and inequities, along with service gaps, have hindered progress, highlighting the need for significant reforms with strong policy implications [1, 6, 9].

Romania’s healthcare system faces significant performance challenges and consistently lags behind most EU countries on key health indicators. Despite some improvements, Romania ranks among the lowest in the EU for life expectancy, infant mortality, health expenditure, and patient satisfaction [1, 10-12]. Life expectancy remains among the lowest in the EU, while avoidable mortality rates are alarmingly high; mortality from treatable causes, specifically, is more than double the EU average, reflecting systemic deficiencies in healthcare delivery [13,14]. For example, life expectancy at birth increased by 1.62 years (from 71.1 in 2000 to 72.8 in 2021), whereas the European average grew by 3.89 years, reaching 76.3 in 2021 [10]. Similarly, infant mortality in Romania decreased markedly from 18 deaths per 1,000 live births in 2000 to 5 per 1,000 in 2020. During the same period, the EU rate also fell from 6 to 3 per 1,000 live births [11]. Furthermore, the rate of preventable deaths ranks third highest in the EU, primarily driven by cardiovascular disease, lung cancer, and alcohol-related harm [12,13]. Chronic underinvestment is widely recognized as a critical underlying factor, with Romania consistently reporting the lowest total health expenditure per capita in the EU (approximately €857 or €1632.2, adjusted for differences in purchasing power, in 2022) [12, 14, 15]. It is important to note, however, that while this PPP adjustment provides a standardized comparison, a general PPP may not fully capture the specific economics of the labor-intensive health sector, warranting a sector-specific analysis [16]. This sustained underfunding manifests in tangible resource shortages, including aging and unevenly distributed hospital infrastructure, as well as difficulties in ensuring adequate supplies of necessary resources, such as equipment and medicines [1, 9, 17]. Finally, persistent governance challenges, notably the widespread perception of informal out-of-pocket payments (‘envelope payments’), continue to erode public trust and hinder system efficiency [18-21].

Romania’s doctor-to-population ratio is lower than the EU average, reflecting a persistent shortage of medical professionals [6]. Romania faces overall workforce shortages (e.g., approximately 3.5 doctors per 1,000 population in 2022, compared to an EU average of nearly 3.9). This shortage is compounded by a severe geographic maldistribution, which particularly disadvantages rural and underserved areas, thereby undermining equitable access to care [22,23]. Compounding these resource issues is a historically hospital-centric service delivery model, resulting in underutilized primary care capacity and inefficient fund allocation, with hospitals consuming the largest share of health spending in the EU (44%) [24]. Addressing these deeply rooted and interconnected challenges requires fundamental structural and paradigm shifts, the focus of the consensus dialogue reported herein.

Reforming Romania’s hospital sector is crucial to addressing performance gaps and creating an environment that fosters both high-quality care and research. After years of significant infrastructure deficits and workforce shortages, many hospitals are unable to satisfy patients’ needs and expectations. Many public hospitals operate in outdated facilities [25], and despite significant new investments from the National Recovery and Resilience Plan (NPRR) to modernize infrastructure [26-28], deep challenges remain. These include improving management, reducing corruption, and stemming the exodus of medical staff [22,29]. Shifting from the current hospital-centric model to an integrated one, where hospitals serve as hubs for complex care, education, and research [30,31], is considered essential for improving outcomes and resilience.

Strengthening clinical research, historically underprioritized, is now a national strategic goal [6,7,32,33]. Romania is increasingly participating in international clinical trials, leveraging its large patient population and aligning with EU regulations [33,34]. This trend is supported by recent pioneering national legislation, such as the 2023 law establishing the right to personalized medicine [35]. Building this ecosystem is seen as crucial for providing patients with access to novel therapies, enhancing clinical skills, and fostering an innovation-friendly environment supported by digital platforms and robust legal frameworks [36-39].

Digital transformation is the critical enabler linking hospital reform and clinical research. Despite recent pushes toward e-health, Romania’s system suffers from pervasive fragmentation, a lack of data interoperability, and a chronically underutilized Electronic Health Record (EHR) [40-43]. These deficiencies create inefficiencies, patient safety risks, and security vulnerabilities [40, 44, 45]. Addressing these gaps through the new National Strategy for Digitalization (2024-2030) and NPRR funding [46-49], while ensuring alignment with the forthcoming European Health Data Space (EHDS), is a cornerstone of the proposed reforms.

The principal aim of this manuscript was to synthesize the collective judgment and consensus recommendations emerging from the multi-stakeholder consensus development conference, “Re-imagining Healthcare: Structural and Paradigm Shifts for a Resilient Future” (Comana, April 2025), aiming to provide an evidence-informed foundation to guide subsequent developments.

## MATERIAL AND METHODS

This study draws on the proceedings of the consensus development conference “Re-imagining Healthcare: Structural and Paradigm Shifts for a Resilient Future,” convened in Comana, Romania, over two and a half consecutive days in April 2025. The conference was organised under the auspices of the Aspen Institute Romania’s Healthcare & Quality of Life Programme and followed the broad principles of the consensus development conference method as described by Halcomb, Davidson, and Hardaker [50]. This approach prioritizes structured, face-to-face deliberation among a deliberately heterogeneous group of stakeholders, facilitating the systematic integration of scientific evidence, professional expertise, and stakeholder perspectives in the formulation of policy-oriented recommendations [51].

### Conference structure

Four pre-defined thematic pillars framed all discussion: (1) Financing the Future of Healthcare; (2) Rethinking Hospital Management and Patient-centred Models; (3) Strengthening Hospital-led Clinical Research; and (4) Artificial Intelligence in Health and the EHDS. Each pillar was introduced by an invited scene-setting presentation that distilled the current evidentiary and policy landscape. Afterward, participants engaged in a sequence of moderated dialogues, small-group breakouts, and plenary debriefs. These interactive formats—characteristic of the Aspen Method—encouraged critical exchange, iterative clarification of viewpoints, and progressive refinement of emerging ideas.

### Participant composition

The seminar convened 31 key stakeholders, including speakers and moderators, representing the principal constituencies in Romania’s healthcare reform landscape. This group comprised high-level representatives from government and public agencies, academic and research institutions, civil society organizations, and the private sector. As detailed in Table 1, the gathering included 17 men and 14 women. While the majority of attendees (*n* = 27) were affiliated with institutions based in Bucharest, the group also included four representatives from other major centers (Târgu Mureș, Cluj-Napoca, Iasi, and Brașov).

### Data generation and documentation

Throughout the conference, designated rapporteurs produced contemporaneous narrative summaries of oral presentations and discussions. Annotated slide decks and written reflections contributed additional documentary material. These artefacts, together with field notes recorded by the facilitators, constitute the primary data corpus for the present manuscript. No formal voting or a priori quantitative threshold for agreement was imposed; instead, convergence of views was sought through open deliberation, iterative paraphrasing, and collective validation during the closing plenary session and during the production of the current list of recommendations. This conversational, discourse-led pathway to consensus is consistent with the epistemological foundations of the consensus development conference method, which emphasizes mutual understanding and negotiated judgment rather than numerical aggregation.

**Table 1:** Profile of seminar participants

Characteristic	Category	*n*
Gender	Male	17
	Female	14
Primary sector	Government & Public Agency	10
	Private Sector (Industry/Finance/Consulting)	10
	Academia & Research	7
	Civil Society (Patient & NGO)	4
Institutional affiliation	Bucharest-based Institution	27
	Regional/International Center (other)	4
Highest academic degree	PhD / MD-PhD	12
	MD (Medical Doctor)	5
	Master’s Degree (MA/MS/MBA/MPH)	10
	Bachelor’s Degree (BA/BS)	4

### Synthesis procedure

After the event, the rapporteur team undertook a rapid, deductive thematic analysis. Textual materials were first organised according to the four pillars; recurrent ideas were then coded and consolidated into higher-order themes. To facilitate this process, we utilized the online platform Miro for collaborative mind-mapping sessions. These virtual workshops enabled the author team to systematically identify, organize, and visually map the key problems, proposed solutions, and emergent sub-themes relevant to each pillar. The organization and final synthesis of the consensus recommendations were conducted using Microsoft Office Excel, which served as a tool for managing the thematic data efficiently and transparently. This structured environment allowed us to consolidate the findings and finalize the six core recommendations presented in the Results section. The resulting analytic memorandum was circulated electronically to all participants within two weeks for member-checking. Minor clarifications suggested during this verification step were incorporated to ensure descriptive accuracy and to reinforce the credibility and confirmability of the synthesis.

### Ethical considerations and rigour

Because the conference constituted a policy-development exercise with professional stakeholders rather than human-subjects research, formal institutional review board approval was not required [52,53]. Nonetheless, all participants were informed that their contributions would inform the synthesized findings, which would be reported in aggregate without individual attribution. Verbal assent for notetaking and the subsequent publication of these synthesized results was obtained from all attendees. Methodological rigour was promoted through (i) triangulation of multiple data sources (oral, written, and visual), (ii) maintenance of a transparent audit trail linking raw notes to analytic themes, and (iii) participant validation of the draft synthesis.

## RESULTS

This section presents the key outcomes and consensus findings from the multi-stakeholder conference. The deliberations, which addressed critical needs in financing, hospital operations, and innovation, converged on a set of six interconnected policy recommendations, which represent the primary findings of this study.

### Synthesis of conference deliberations

The recommendations resulted from intensive, structured deliberations guided by four thematic pillars. While collectively agreed upon, the process revealed key focus areas and debates. Pillar 1, "Financing the Future of Healthcare," reached consensus on the unsustainability of current financing, emphasizing the need to broaden the contribution base and mandate multi-year budgets as prerequisites for reforms. Pillar 2, "Rethinking Hospital Management and Patient-Centered Models," sparked extensive discussion on dismantling the entrenched physician-centered model and addressing inefficiencies, inequities, and informal payments. The complex idea of regulated dual practice was explored as a pragmatic tool, with concerns over its implementation before full patient-centered reform. Pillar 3, "Strengthening Hospital-Led Clinical Research," highlighted Romania’s underperformance in clinical trials, focusing on shortages of specialized personnel, rebuilding sponsor trust, and institutionalizing research. Site Management Organizations were discussed as a structural solution. Pillar 4, "AI and the European Health Data Space," emphasized digital transformation and reached consensus that interoperability is crucial. Challenges include data fragmentation, regulatory alignment with EHDS, and ethical and practical issues of AI in clinical practice. The consensus recommendations detailed in the following section were the product of intensive, structured deliberations guided by the four thematic pillars. While the final recommendations represent the collective convergence of views, the process itself revealed several key areas of focus and debate that informed the synthesis.

### The six consensus recommendations

The six core recommendations synthesized from the conference deliberations are detailed below and visually summarized in Table 2.

**Table 2:** Consensus recommendations for the Romanian healthcare reform

Conference pillar	Primary recommendation(s)
**Pillar 1: Financing the future of healthcare**	Rec. 1: Re-engineering NUSSHIF revenue and budgeting Rec. 3: Establishing equitable hospital reimbursement Rec. 6: Optimizing resource allocation
**Pillar 2: Rethinking hospital management**	Rec. 2: Mandating the paradigm shift to patient-centered care Rec. 6: Carefully introducing regulated dual practice
**Pillar 3: Strengthening hospital-led clinical research**	Rec. 5: Strategically developing a competitive clinical trials ecosystem
**Pillar 4: AI and the European health data space**	Rec. 4: Accelerating national digital health transformation & EHDS alignment

### Recommendation 1: Re-engineering NUSSHIF revenue and budgeting for stability and predictability

**Figure 1:**
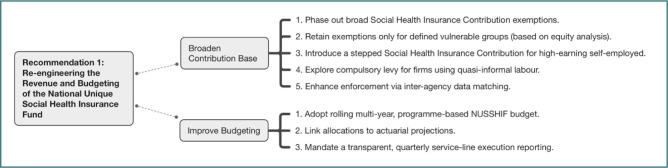
Key actions for Recommendation 1: NUSSHIF revenue and budgeting reform

Following successive legislative changes exempting large groups (such as pensioners and specific sectors), the contributor base had plummeted to extremely low levels (around 27% previously); although recovering slightly to 35% of residents by 2023, this figure remains profoundly insufficient, especially when compounded by an estimated two million or more uninsured individuals and over ten million exempt beneficiaries. Meanwhile, supporting over ten million exempt beneficiaries was deemed patently unsustainable [24]. Participants firmly agreed that the National Unique Social Health Insurance Fund’s (NUSSHIF) financial viability is critically undermined by its revenue structure. Therefore, consensus strongly supported a phased and deliberate withdrawal of broad state social insurance contributions exemptions, particularly those enacted after 2017, while ensuring continued protection for clearly defined vulnerable groups through transparent criteria. Complementary measures considered essential included introducing stepped state social insurance contribution schedules for high-earning self-employed individuals and exploring mechanisms such as a compulsory levy for firms potentially leveraging quasi-informal labour, with enforcement bolstered by enhanced real-time data matching across the NHIH, the National Agency for Fiscal Administration, and labour inspectorates. Concurrently, participants criticized the chronic unpredictability of state balancing subsidies, which severely hampers NHIH’s strategic planning capacity, and called unequivocally for adopting a rolling multi-year, programme-based NUSSHIF budget. This framework must link allocations to updated actuarial projections and mandate transparent, quarterly publication of detailed service-line execution data to foster accountability and enable informed analysis. Rapidly enhance public health data collection, integration, and analytic capacities in healthcare authorities, and promote data exchange for data-based decision making. The key actions for this recommendation are outlined in Figure 1.

### Recommendation 2: Mandating the paradigm shift to patient-centered hospital care

**Figure 2:**
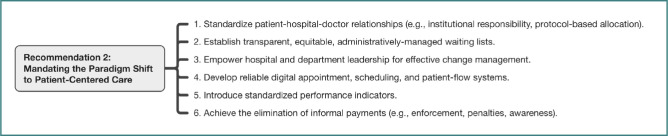
Key actions for Recommendation 2: Mandating the patient-centered hospital paradigm

A central point of convergence was the identification of the prevailing physician-centered operational model in most Romanian public hospitals as a root cause of numerous systemic failures. This deeply ingrained paradigm, a legacy, participants noted, partly inherited from the communist era and solidified post-1990 when personal relationships often became the de facto mechanism for navigating access and securing care within a weakly structured public system, manifests in patients forming direct, frequently informal, social capital with specific doctors, bypassing institutional pathways; where access is dictated by personal connections and opaque, physician-held waiting lists; where professional duties are blurred by extensive unpaid overtime dedicated to ‘personal’ patients; and where teamwork is undermined by internal competition. This model was recognized as severely impacting patient accessibility, quality, and continuity of care, as well as multidisciplinary collaboration, inter-facility transfers, resident training, meritocratic advancement, and departmental management. Participants also noted that it inherently fosters an environment conducive to illegal, informal payments. Despite paradoxically keeping the chronically underfunded system afloat through immense individual effort, participants firmly concluded that a fundamental paradigm shift towards patient-centered hospital care is non-negotiable for meaningful progress. This shift necessitates the hospital, as an institution, taking primary responsibility for the patient journey. Achieving this demands the mandatory implementation of several interconnected preconditions: formally normalizing the patient-hospital-doctor relationship (with patients assigned to attending physicians institutionally by department heads based on clear protocols); establishing equitable, transparent, administratively-managed departmental waiting lists based on objective medical criteria; decisively empowering hospital management and department heads with the authority and accountability to lead and enforce this transformation; implementing robust digital appointment, scheduling, and patient-flow systems; introducing standardized performance and activity indicators after organizational normalization to monitor quality and efficiency, not just volume; and pursuing the absolute eradication of all informal payments through unwavering enforcement, significant penalties, and sustained public awareness campaigns. The interconnected preconditions for this paradigm shift are illustrated in Figure 2.

**Figure 3:**
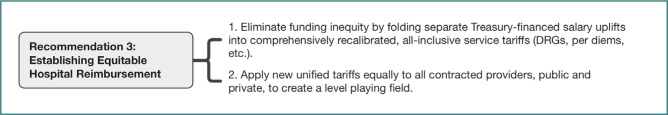
Key action for Recommendation 3: Establishing equitable hospital reimbursement

### Recommendation 3: Establishing equitable hospital reimbursement as an enabler

Participants agreed that financial mechanisms must actively support, not hinder, the hospital paradigm shift. The immediate, critical step identified is to eliminate the profound inequity in hospital funding by folding the significant, separate Treasury-financed salary uplifts (currently benefiting only public hospitals) into comprehensively recalibrated, all-inclusive service tariffs (DRGs, chronic care per diems, etc.) that apply equally to all contracted providers, public and private. While acknowledging potential implementation challenges, such as managing staff expectations and addressing possible union concerns about changes to remuneration structures, this measure was deemed essential to eliminate the current detrimental asymmetry in which public facilities receive dual funding streams. In contrast, private ones often require co-payments for equivalent services, thereby creating a level playing field where funding genuinely follows the patient and reflects the complexity of services rendered, regardless of ownership. The core mechanism for this recommendation is shown in Figure 3.

### Recommendation 4: Accelerating national digital health transformation and EHDS alignment

**Figure 4:**
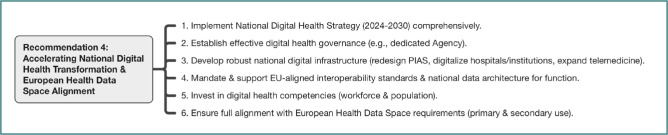
Key actions for Recommendation 4: Digital health transformation and EHDS alignment

Recognizing that digital transformation underpins progress across all areas, participants strongly endorsed the swift and comprehensive implementation of the National Digital Health Strategy (2024-2030). The consensus highlighted the need to prioritize key actions and leverage the PNRR and other funding sources. This includes establishing effective digital health governance, potentially via the proposed dedicated agency, to ensure coordination and consistent policy implementation. Developing a robust and secure national digital health infrastructure is paramount, focusing on redesigning core systems (such as PIAS -the health insurance IT platform), digitalizing institutions and hospitals, and expanding telemedicine services. Critically, the adoption of EU-aligned interoperability standards (e.g., FHIR, HL7, SNOMED-CT) and a national health data architecture must be mandated and supported to enable seamless data exchange and realize a functional national EHR. In the future, eHealth Digital Service Infrastructure (eHDSI) will be enabled through the establishment and operation of National Contact Points for eHealth (NCPeH) at the level of each EU Member State for the exchange of health data and the provision of cross-border ePrescription/eDispensation and Patient Summary services. Significant investment is required to develop digital health competencies across both the workforce and the general population through updated training and accessible literacy programs. Finally, all national digitalization efforts must ensure complete alignment with the EHDS requirements and timelines, preparing Romanian systems for both primary and secondary cross-border data use. The strategic components of this recommendation are detailed in Figure 4.

### Recommendation 5: Strategically developing a competitive clinical trials ecosystem

**Figure 5:**
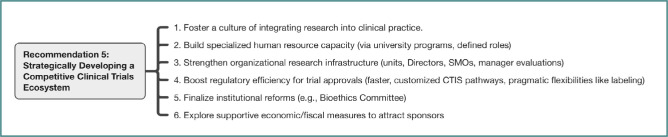
Key actions for Recommendation 5: Clinical trials ecosystem development

Given Romania’s lagging health outcomes and its significant underperformance in clinical research, participants strongly recommended elevating the development of a robust clinical trials ecosystem to a national strategic priority. This was framed as vital not only for improving patient access to potentially life-saving innovative therapies but also for advancing medical knowledge, enhancing the quality of care within participating institutions, and generating significant economic value. Key recommendations focused on: fostering a culture where research is integral to clinical practice; systematically building specialized human resource capacity (addressing documented shortages of clinical pharmacists, study nurses, data managers, coordinators) through university-led initiatives like dedicated Master’s programs and clearly defined hospital roles; strengthening organizational research infrastructure within hospitals (reactivating dedicated units, empowering Research Directors, utilizing Site Management Organizations (SMOs), including research activity in manager evaluations); significantly boosting regulatory efficiency and predictability by adopting faster, customized approval pathways within the EU CTIS for mono-national/early-phase trials (aiming for <60-day or even 30-day targets, adopting pragmatic flexibilities like English-language labeling allowances following international precedents, and streamlining amendment approvals); finalizing critical institutional reforms (like the Bioethics Committee’s transfer and reorganization); and actively exploring supportive economic and fiscal measures to attract sponsors and investment, mitigating current deterrents related to drug pricing and reimbursement policies which currently limit Romania’s attractiveness primarily to late-phase "back-up" scenarios. The multi-pronged strategy for this recommendation is visualized in Figure 5.

### Recommendation 6: Optimizing resource allocation and carefully introducing regulated dual practice

Alongside structural reforms, consensus affirmed the need to strategically reallocate stabilized NUSSHIF to historically underfunded areas, such as primary care and prevention, and to explicitly link disbursements to measurable performance indicators. Managing pharmaceutical expenditures requires maintaining the claw-back mechanism but enhancing its predictability (e.g., indexing the reference value) and significantly expanding the use of value-based contracts for high-cost medicines. Piloting efficiency drivers, such as tightened prescribing protocols for low-value diagnostics and alternative payment models (e.g., global budgets with gainsharing), in high-cost specialties (e.g., oncology, dialysis) was also strongly supported.

**Figure 6:**
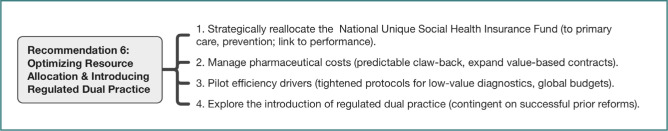
Key actions for Recommendation 6: Resource allocation and regulated dual practice

Within this comprehensively reformed landscape, specifically contingent upon the successful implementation of the patient-centered paradigm, robust performance metrics, and the complete elimination of informal payments, participants endorsed exploring regulated dual practice (defined as private activity in public hospitals outside core hours for a formal fee) as a pragmatic transitional tool. Its potential benefits—providing a transparent avenue for patient choice, aiding retention of high-performing physicians with supplementary legal income, and generating direct revenue for hospitals—were seen as valuable for managing the difficult shift away from the deeply entrenched informal system, provided it is strictly governed and does not compromise public sector duties. To embrace the anticipatory nature of this reform, we must proactively identify and develop mitigation strategies for potential risks related to regulated dual practice. Beyond concerns about inequities in access to care, other downsides include deprioritized public-sector duties due to physician fatigue, diverted public resources to support private activities when cost accounting is insufficient, ‘cherry-picking’ less complex cases for private slots, and new governance complexities that may inadvertently allow informal practices to persist. Addressing these risks underscores the need for robust, transparent, and actively enforced regulatory frameworks, along with continuous monitoring, to ensure that dual practice serves its intended purpose without compromising public health objectives. The specific actions for this recommendation are presented in Figure 6.

## DISCUSSION

The consensus recommendations synthesized in this study present a direct and comprehensive response to the deep-seated performance challenges that have historically constrained the Romanian healthcare system [1, 6, 9]. The findings present a multi-pronged strategy designed to address the core deficiencies in financing, service delivery, and innovation identified at the outset of the manuscript.

### Analysis of implementation challenges

While the consensus recommendations provide a clear roadmap, participants also identified formidable, multifaceted implementation challenges. These challenges, detailed below, require exceptional leadership, sustained political will, significant investment, and proactive management.

### Navigating the profound hospital paradigm shift implementation

The transition away from the deeply ingrained physician-centered culture towards institutional responsibility and patient-centered care was unanimously recognized as the most significant, complex, and potentially contentious long-term challenge. Participants anticipate a complex, protracted process, likely involving initial resistance from stakeholders accustomed to the old ways. A critical hurdle will be managing the inevitable temporary negative consequences as informal coping mechanisms are dismantled, potentially including initial increases in formal waiting times, heightened visibility of resource shortages previously masked by individual effort, a risk of defensive medical practices, and potential demotivation or migration of staff who are unwilling or unable to adapt. Exceptional change management capabilities within hospitals, along with clear and consistent public communication that explains the rationale and timeline, are paramount. Addressing the complex ethical considerations surrounding the introduction of regulated dual practice fees, ensuring fairness and transparency regarding differential access within the public system based on ability to pay, requires careful policy design and societal dialogue.

### Overcoming systemic barriers to digital health adoption

Achieving national digital health goals faces substantial hurdles, including addressing the critical shortage of specialized digital health personnel across the system, combating negative public and professional perceptions about data use, and developing the robust digital infrastructure necessary for widespread implementation, including truly interoperable EHRs, functional registries, and an electronic health data system. Ensuring sustained regulatory agility and efficiency, specifically for digital health tools, is also critical for fostering innovation and adoption. A significant challenge highlighted is ensuring that the push for digitalization does not exacerbate existing health inequities. Proactive measures are necessary to ensure equitable access to digital tools and services for all citizens, regardless of their location, income, or level of digital literacy. This requires targeted investment in infrastructure for underserved areas, as well as specific programs to enhance digital health literacy among vulnerable populations and the workforce.

### Addressing obstacles to clinical research revitalization

Achieving Romania’s potential as a competitive location for clinical trials necessitates overcoming substantial existing barriers identified in the discussions. These include: tackling the critical shortage of specialized research personnel through sustained educational investment; actively combating negative public and professional perceptions regarding trial participation; developing robust national infrastructure for patient identification and recruitment, including broader access to genetic testing and networked eligibility assessments; upgrading physical research facilities within hospitals; systematically rebuilding sponsor trust eroded during previous periods of regulatory inconsistency, for instance, by enhancing transparency and predictability through concrete measures such as the establishment of a public portal for clinical trial approval statuses and timelines; and ensuring continuous improvement and sustained efficiency in regulatory approval processes beyond initial reforms. The sheer breadth and interconnected nature of the challenges Romania aims to resolve simultaneously—ranging from fundamental infrastructure and personnel deficits to rebuilding systemic trust and ensuring enduring regulatory agility—highlights a particularly ambitious national commitment to substantially elevate its clinical research standing within the European context. The success of this strategy, participants stressed, is fundamentally dependent on the successful implementation of the broader financial and hospital system reforms.

### Addressing interconnected political, technical, and fiscal complexities

Enacting reforms, such as changes to the state social insurance contributions, directly impacts politically sensitive constituencies, requiring careful sequencing, transparent equity analyses, and firm political resolve. The envisioned advancements in budgeting, performance monitoring, hospital management systems, and clinical trial administration all depend critically on overcoming significant technical hurdles related to IT system interoperability, data standardization, and digital infrastructure development, which requires substantial investment —potentially leveraging PNRR funds —to achieve goals such as an EHDS-compatible health data ecosystem. Furthermore, the healthcare system’s long-term fiscal sustainability remains under pressure from demographic trends and wage demands, reinforcing the need for rigorous financial stress-testing of reform impacts and potentially codifying automatic stabilization rules for state subsidies to mitigate funding volatility. Defining the precise legislative framework and operational regulations for dual practice, as well as their interaction with complementary private health insurance, also represents a significant task ahead.

### Ensuring effective governance, implementation fidelity, and sustained momentum

Translating this complex web of policies into tangible, lasting improvements demands robust governance structures and vigilant monitoring. Managing potential unintended consequences of specific measures requires adaptive implementation frameworks. Crucially, to prevent reform stagnation or a return to past ad hoc practices, participants strongly reiterated the consensus call to establish an independent, legally empowered Health Financing Oversight Board. This body, vested with clear audit and corrective action capabilities, is seen as essential for ensuring implementation fidelity across all reform pillars, maintaining accountability, fostering transparency, and championing the strategic vision for a modernized, equitable, and patient-centered Romanian healthcare system over the long haul.

### Contextualizing the recommendations

The proposed reforms to stabilize the National Unique Social Health Insurance Fund through a broadened contributor base and predictable multi-year budgeting directly address the critical issue of chronic underinvestment, which has left Romania with the lowest per capita health expenditure in the EU [12, 14, 15]. This financial instability has previously been identified as a key source of resource shortages, ranging from infrastructure to medicines [1, 9, 17].

Similarly, the call for a mandatory paradigm shift to patient-centered hospital care targets the dual problems of an inefficient, hospital-centric model [24] and the pervasive issue of informal "envelope payments" that erode public trust [18, 19, 21]. This structural change, combined with equitable reimbursement models, seeks to build the institutional accountability necessary to overcome decades-old governance challenges that have hindered progress since the post-1989 transition [1-4].

This digital fragmentation, as noted in the introduction, has far-reaching consequences. They manifest as healthcare system inefficiencies, such as data duplication and compromised patient safety [40]. For instance, the potential for data errors in the EHR due to insufficient training has been noted [54], and an alarmingly low 5% of people with chronic conditions in Romania are managed in primary care practices that can exchange medical records electronically, a stark contrast to the OECD PaRIS average of 57% [43]. Furthermore, this fragmentation severely hampers public health surveillance, research, and the formulation of evidence-based health policies [7]. The often outdated IT infrastructure also presents significant data security vulnerabilities, starkly illustrated by recent ransomware attacks on numerous hospitals, and complicates effective GDPR compliance [44,45]. Much of today’s medical data in Romania (as elsewhere) remains siloed and incompatible across facilities, which hinders progress in both clinical care and research [46].

Furthermore, the strategic priorities of accelerating digital transformation and revitalizing the clinical trials ecosystem are positioned as essential levers to combat Romania’s alarmingly high rates of treatable and preventable mortality [12,13]. By aligning with the European Health Data Space (EHDS) and fostering access to innovative therapies, these recommendations aim to fulfill the goals of successive national health strategies that have long identified e-health and research as priorities [5-8]. Finally, the recommendations to reallocate resources toward primary care and cautiously explore regulated dual practice offer pragmatic approaches to mitigate the persistent challenge of workforce shortages and the geographic maldistribution of medical professionals [22,23].

Integrating clinical trials and health innovation is fundamental, and formalizing this synergy within a life sciences strategy for healthcare systems promises to significantly accelerate medical progress. The evolution of clinical trials, increasingly powered by digital technologies, AI, and innovative designs such as in silico trials, enables faster development and implementation of novel therapies [55-58]. Such strategic integration is key to delivering long-term societal value. This is achieved by improving patient outcomes through the rapid adoption of evidence-based, personalized care [59,60], boosting healthcare efficiency via streamlined operations and collaborative networks [37, 55, 61], and fostering a more resilient, innovation-driven economy by attracting investment and spurring technological development [37,56,60,61]. Ultimately, clinical trials and health innovation go hand in hand, and embedding clinical research into a life sciences strategy for the healthcare system would not only accelerate medical progress but also deliver long-term societal value by improving patient outcomes, boosting healthcare efficiency, and fostering a more resilient, innovation-driven economy.

A unified and interoperable digital infrastructure will vastly improve data availability for public health and research. It will enable the routine collection of health indicators and outcomes, helping identify gaps and monitor interventions. Crucially, Romania’s digital transformation is closely aligned with EU-wide trends, especially the upcoming European Health Data Space (EHDS). The EHDS is a cornerstone initiative of the European Health Union [62], establishing a common framework for sharing electronic health data across member states. Under the EHDS regulation, individuals will be empowered to access and share their health records across borders, and a harmonized technical and legal standard for EHRs will facilitate interoperability and the secondary use of health data for research and policy [62]. This EU legislation, which is set to enter into force in 2025, is highly relevant for Romania, as it provides both impetus and guidance for the country’s national digitalization efforts. By building interoperable systems now, Romania not only improves internal efficiency but also positions itself to participate in cross-border research and innovation initiatives.

Currently, the National Strategy for Digitalization in Health 2024-2030 [46], along with substantial investments via the PNRR, provides renewed impetus. The PNRR allocates significant funding (some analyses cite approximately €442 million for overall healthcare digitalization [4]), with specific calls targeting health system components, to establish an integrated e-Health system that connects numerous healthcare providers and expands telemedicine services [47-49]. This includes optimizing the core Health Insurance IT Platform (PIAS), which encompasses the EHR and e-Prescription systems, digitalizing health institutions, as well as enhancing hospital IT infrastructure [63]. Specific projects such as eDES for expanding EHR connectivity [42], and RegInterMed for developing national health registries with an emphasis on international standards like SNOMED CT and LOINC are also underway [64]. The establishment of new coordinating bodies, such as the National Agency for Infrastructure Development in Health (ANDIS) [18], is also intended to bolster these efforts [65]. However, the success of these initiatives critically depends on overcoming historical deficiencies in governance—Romania notably lacks a dedicated, empowered structure for overall digital health coordination—and on the rigorous development, adoption, and enforcement of a comprehensive national health data interoperability framework.

In essence, this study documents stakeholder consensus that is not a set of isolated fixes but an interconnected roadmap in which financial stability enables structural reform, and digital innovation underpins improvements in both care quality and research capacity. While the path to implementation is formidable, the alignment between the identified problems and the proposed solutions provides a coherent foundation for transformative change.

### Strengths and limitations of the study

A primary strength of this study is its consensus methodology, which secured multi-stakeholder agreement on a comprehensive reform package. This provides policymakers with a degree of "political cover" to pursue difficult but necessary changes, as a broad coalition of experts from government, industry, academia, and civil society legitimizes the recommendations. The interconnected, systems-level approach is another key strength, acknowledging that isolated fixes are unlikely to succeed.

However, the study has significant limitations. First, the consensus method, by its nature, produces a unified narrative that submerges stakeholder conflict. The final recommendations do not accurately reflect the complex negotiations and dissenting views inherent in any multi-stakeholder process. This limits our understanding of the relative political weight behind each proposal. Second, the study is prescriptive but not operational; it defines *what* must be done, but does not provide a detailed, sequenced blueprint for implementing these complex reforms. Third, while including patient-advocacy organizations, the conference was a professional exercise, and the direct voice of a broader patient population was not a primary data source for defining patient-centeredness. Finally, the scope is intentionally focused on the Romanian context, and while its principles are valuable, the specific recommendations are not directly generalizable without significant adaptation.

## CONCLUSION

The consensus development conference yielded a clear and compelling mandate: profound, systemic, and interconnected reforms are required to address Romania’s deep-seated healthcare challenges. Participants collectively affirmed that incremental adjustments are insufficient and that maintaining the status quo is untenable, risking further degradation of the system.

The consensus detailed in the results underscores the critical interdependence of these reform pillars. Stabilizing the National Health Insurance Fund is not merely a fiscal exercise but the essential foundation for meaningful improvements in service delivery and innovation. Similarly, the proposed paradigm shift toward patient-centered, institutionally accountable hospital care is fundamental to enhancing quality and creating an environment where clinical research and innovative mechanisms such as regulated dual practice can thrive.

Acknowledging the formidable implementation challenges ahead, the dialogue affirmed that translating this consensus into tangible reality demands sustained political commitment, broad multi-stakeholder collaboration, and robust governance. This represents the necessary journey to achieve a transformed Romanian healthcare system: one that is financially sustainable, institutionally accountable, equitable in access, and capable of fostering innovation and motivating its health workforce.

## Data Availability

The primary data supporting the conclusions of this manuscript are the synthesized proceedings and recommendations from the “Reimagining Healthcare: Structural and Paradigm Shifts for a Resilient Future” conference. These findings are in this article. The raw data, such as narrative summaries, annotated slide decks, and facilitators’ notes, are not publicly available to protect participant confidentiality and the nature of the consensus development process, as informed consent was obtained for aggregate reporting without individual attribution. Inquiries regarding source materials can be directed to the corresponding author upon reasonable request, ensuring participant confidentiality is maintained.

## Personal thanks

We extend our gratitude to the stakeholders who generously contributed their time and expertise to this research. The authors wish to express their sincere gratitude to the Aspen Institute Romania for organizing and hosting the “Reimagining Healthcare: Structural and Paradigm Shifts for a Resilient Future” consensus development conference in Comana, April 2025. The Institute’s Healthcare & Quality of Life Programme provided the essential platform and facilitative environment that enabled the rich discussions and the synthesis of recommendations presented in this manuscript.
